# Supplementary material for: Population-specific positive selection on low CR1 expression in malaria-endemic regions
Source: PLoS One. 2023 Jan 10;18(1):e0280282. doi: 10.1371/journal.pone.0280282 (PMC9831336; doi:10.1371/journal.pone.0280282)
Supplement: S1 Fig — Graphical annotation of the structure of the CR1 locus at different levels. Location of the CR1 gene on chromosome 1 is indicated by a red vertical line (UCSC genome browser top image). The genome annotation represents the structure of the two major CR1 isoforms H and L with the introns as blue arrows and exons as vertical blue lines. Black arrows indicate the locations on the CR1 gene of the four SNPs which can determine expression levels (rs73689510 on exon 19, rs2274567 on exon 22, rs11118133 on intron 27 and rs3811381 on exon 33) and of a SNP which can affect ESR (rs12034598 on intron 24). The locations and orientations of the Low‐Copy Repeats (LCRs) are represented as horizontal arrows below the genome annotation while absence of a particular LCR is indicated as a deletion (white rectangular box). The transcript annotation represents the number of exons (boxes) in each isoform. Each LCR consists of 8 exons and their locations on the genome is highlighted by shaded areas. The protein annotation shows the organization of the long homologous repeat regions (LHRs). Cylinders of different colours represent different LHRs. The white box labelled TM denotes the region encoding the transmembrane domain. In addition, the protein level depicts the CR1 functional domains with each circle representing a separate short consensus repeat (SCR). Each LHR is in fact composed by 7 different SCRs. The longer isoform H possess an additional LHR S not found in the shorter isoform which can increase the number of binding sites to the complement proteins (white spheres and ovals). Darker coloured spheres at the protein level represent SCRs involved in the binding to the complement and to malaria parasite proteins (grey and black ovals). White empty spheres indicate the locations of the Knops blood group antigen erythrocyte polymorphisms. (PDF) [file pone.0280282.s001.pdf]

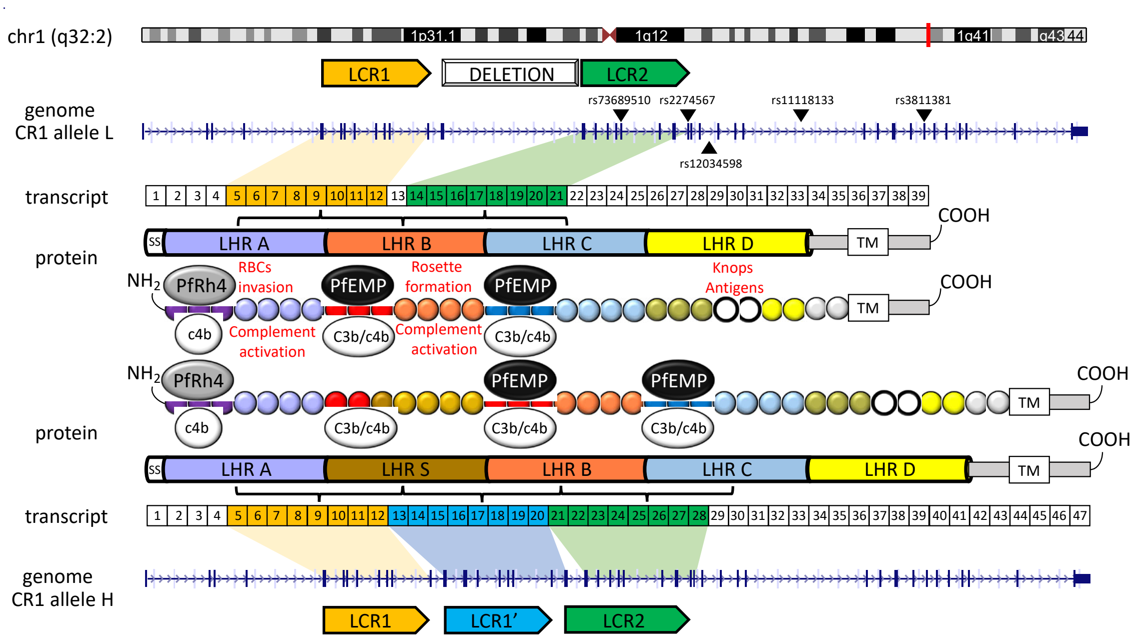

S1 Fig. CR1 protein structure. CR1 protein structure. Graphical annotation of the structure of the CR1 locus at different levels. Location of the CR1 gene on chromosome 1 is indicated by a red vertical line (UCSC genome browser top image). The genome annotation represents the structure of the two major CR1 isoforms H and L with the introns as blue arrows and exons as vertical blue lines. Black arrows indicate the locations on the CR1 gene of the four SNPs which can determine expression levels (rs73689510 on exon 19, rs2274567 on exon 22, rs11118133 on intron 27 and rs3811381 on exon 33) and of a SNP which can affect ESR (rs12034598 on intron 24). The locations and orientations of the Low-Copy Repeats (LCRs) are represented as horizontal arrows below the genome annotation while absence of a particular LCR is indicated as a deletion (white rectangular box). The transcript annotation represents the number of exons (boxes) in each isoform. Each LCR consists of 8 exons and their locations on the genome is highlighted by shaded areas. The protein annotation shows the organization of the long homologous repeat regions (LHRs). Cylinders of different colours represent different LHRs. The white box labelled TM denotes the region encoding the transmembrane domain. In addition, the protein level depicts the CR1 functional domains with each circle representing a separate short consensus repeat (SCR). Each LHR is in fact composed by 7 different SCRs. The longer isoform H possess an additional LHR S not found in the shorter isoform which can increase the number of binding sites to the complement proteins (white spheres and ovals). Darker coloured spheres at the protein level represent SCRs involved in the binding to the complement and also to malaria parasite proteins (grey and black ovals). White empty spheres indicate the locations of the Knops blood group antigen erythrocyte polymorphisms.
