# Supplementary material for: Population-specific positive selection on low CR1 expression in malaria-endemic regions
Source: PLoS One. 2023 Jan 10;18(1):e0280282. doi: 10.1371/journal.pone.0280282 (PMC9831336; doi:10.1371/journal.pone.0280282)
Supplement: S2 Fig — The top 10% most negative values are plotted in the CR1 gene region including 50kb upstream and downstream for each of the 11 population groups analysed. Dots and triangles represent SNPs having percentile ranking values equal or lower then 0.10 (< 10%) indicated on the Y axis over the location on chromosome 1 (X axis) in Mega bases (Mb). The green bar under the X axis represents the CR1 gene region, and the mesh area indicates repeats. The regions 50kb upstream and downstream of the CR1 gene are indicated as a line. In the DNA repeat region, no SNPs were called. In addition, purple triangles indicate the locations of rs2274567 exon 22, rs12034598 intron 24 and rs3811381 exon 33 SNPs. (PDF) [file pone.0280282.s002.pdf]

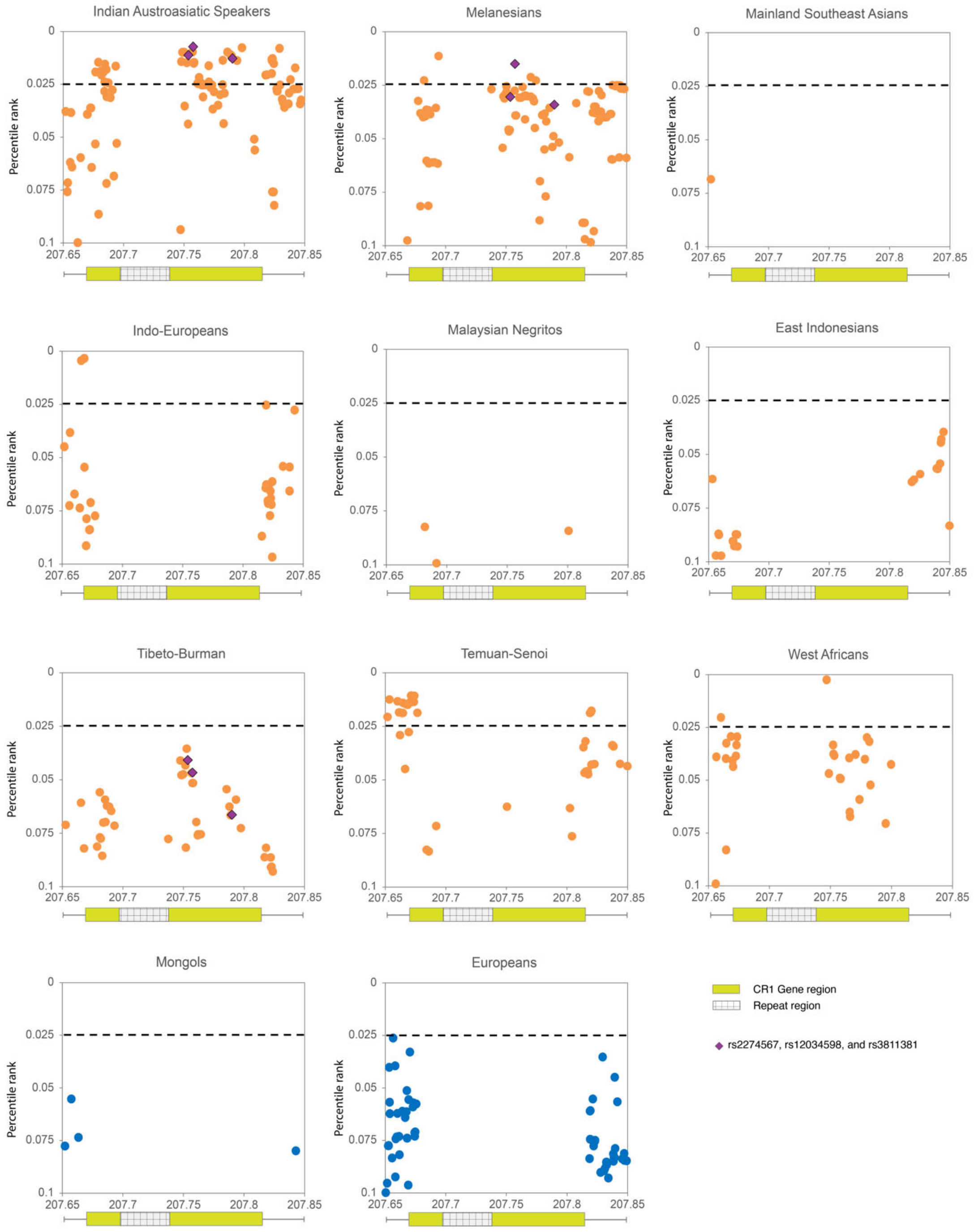

S2 Fig. Genome-wide percentile ranking of the standardised iHS negative values. The top 10% most negative values are plotted in the CR1 gene region including 50kb upstream and downstream for each of the 11 population groups analysed. Dots and triangles represent SNPs having percentile ranking values equal or lower then 0.10 (< 10%) indicated on the Y axis over the location on chromosome 1 (X axis) in Mega bases (Mb). The green bar under the X axis represents the CR1 gene region, and the mesh area indicates repeats. The regions 50kb upstream and downstream of the CR1 gene are indicated as a line. In the DNA repeat region, no SNPs were called. In addition, purple triangles indicate the locations of rs2274567 exon 22, rs12034598 intron 24 and rs3811381 exon 33 SNPs.
