# Supplementary material for: Population-specific positive selection on low CR1 expression in malaria-endemic regions
Source: PLoS One. 2023 Jan 10;18(1):e0280282. doi: 10.1371/journal.pone.0280282 (PMC9831336; doi:10.1371/journal.pone.0280282)
Supplement: S3 Fig — The top 10% most positive are plotted in the CR1 gene region including 50kb upstream and downstream for each of the 11 population groups analysed. Dots and triangles represent SNPs having percentile ranking values equal or lower then 0.10 (< 10%) indicated on the Y axis over the location on chromosome 1 (X axis) in Mega bases (Mb). The green bar under the X axis represents the CR1 gene region, and the mesh area indicates repeats. The regions 50kb upstream and downstream of the CR1 gene are indicated as a line. In the DNA repeat region, no SNPs were called. In addition, dark green triangles indicate the locations of SNPs showing significantly low expression levels of CR1 in brain tissues (S10 Fig): rs3886100, rs11803956, rs12041437, rs17186848, rs12034383, and rs11803366. (PDF) [file pone.0280282.s003.pdf]

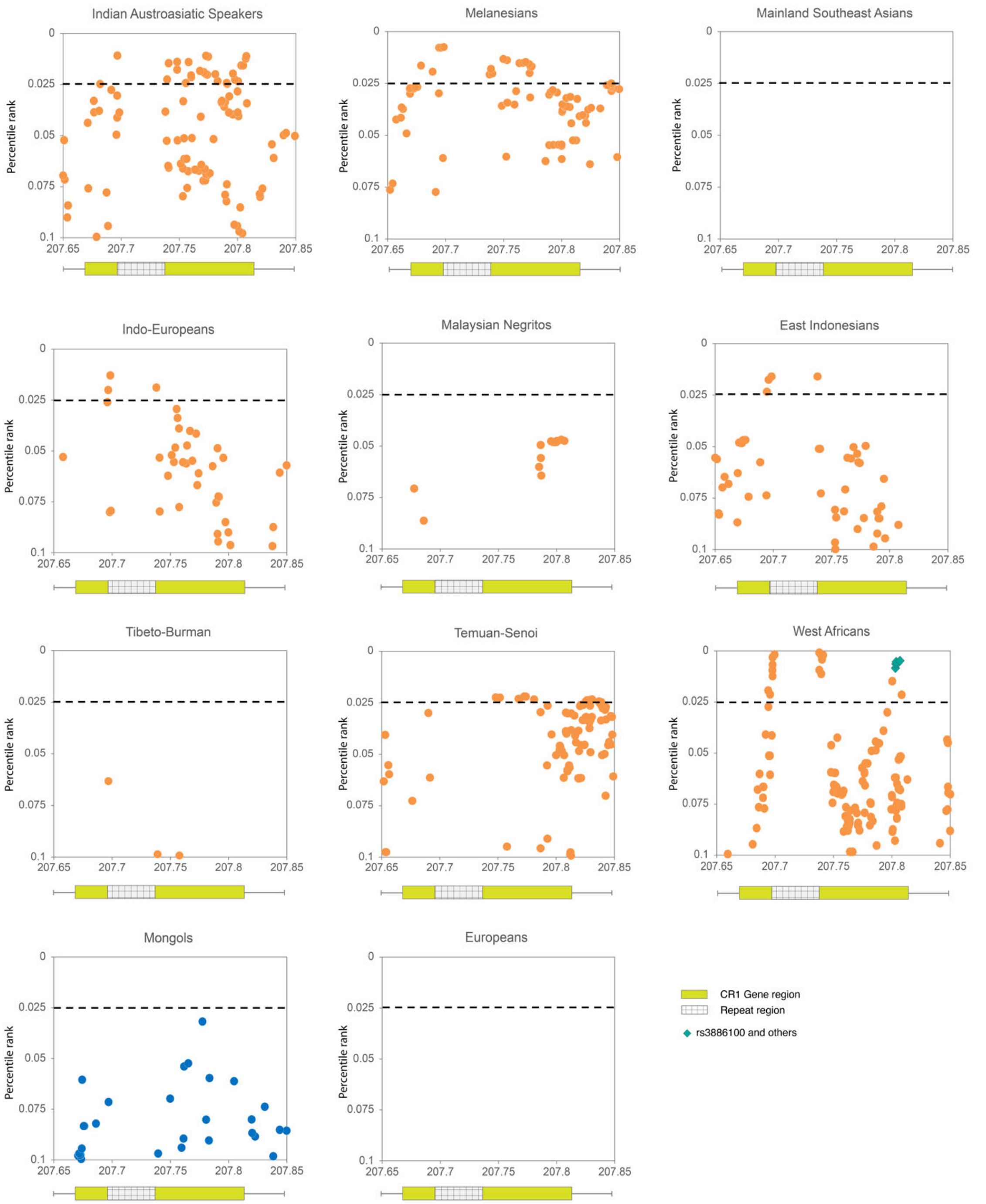

S3 Fig. Genome-wide percentile ranking of the standardised iHS positive values. The top 10% most positive are plotted in the CR1 gene region including 50kb upstream and downstream for each of the 11 population groups analysed. Dots and triangles represent SNPs having percentile ranking values equal or lower then 0.10 (< 10%) indicated on the Y axis over the location on chromosome 1 (X axis) in Mega bases (Mb). The green bar under the X axis represents the CR1 gene region, and the mesh area indicates repeats. The regions 50kb upstream and downstream of the CR1 gene are indicated as a line. In the DNA repeat region, no SNPs were called. In addition, dark green triangles indicate the locations of SNPs showing significantly low expression levels of CR1 in brain tissues (Figure S10): rs3886100, rs11803956, rs12041437, rs17186848, rs12034383, and rs11803366.
