# Supplementary material for: Population-specific positive selection on low CR1 expression in malaria-endemic regions
Source: PLoS One. 2023 Jan 10;18(1):e0280282. doi: 10.1371/journal.pone.0280282 (PMC9831336; doi:10.1371/journal.pone.0280282)
Supplement: S4 Fig — The XP‐EHH values are plotted in the CR1 gene region including 50kb upstream and downstream for each of the 9 endemic population groups compared to the reference population of Mongols (non-endemic). Dots and triangles represent SNPs having percentile ranking values equal or lower then 0.10 (top 10%) indicated on the Y axis over the location of SNPs on chromosome 1 (X axis) in Mega bases (Mb). The green bar under the X axis represents the CR1 gene region, and the mesh area indicates repeats. The regions 50kb upstream and downstream of the CR1 gene are indicated as a line. In the DNA repeat region no SNPs called. We detected a larger number of SNPs having a percentile ranking of top 5% in the two endemic population groups (Indian Austroasiatic, Melanesians, and West Africans) in the CR1 gene region. Purple triangles indicate rs2274567, rs12034598, and rs3811381. (PDF) [file pone.0280282.s004.pdf]

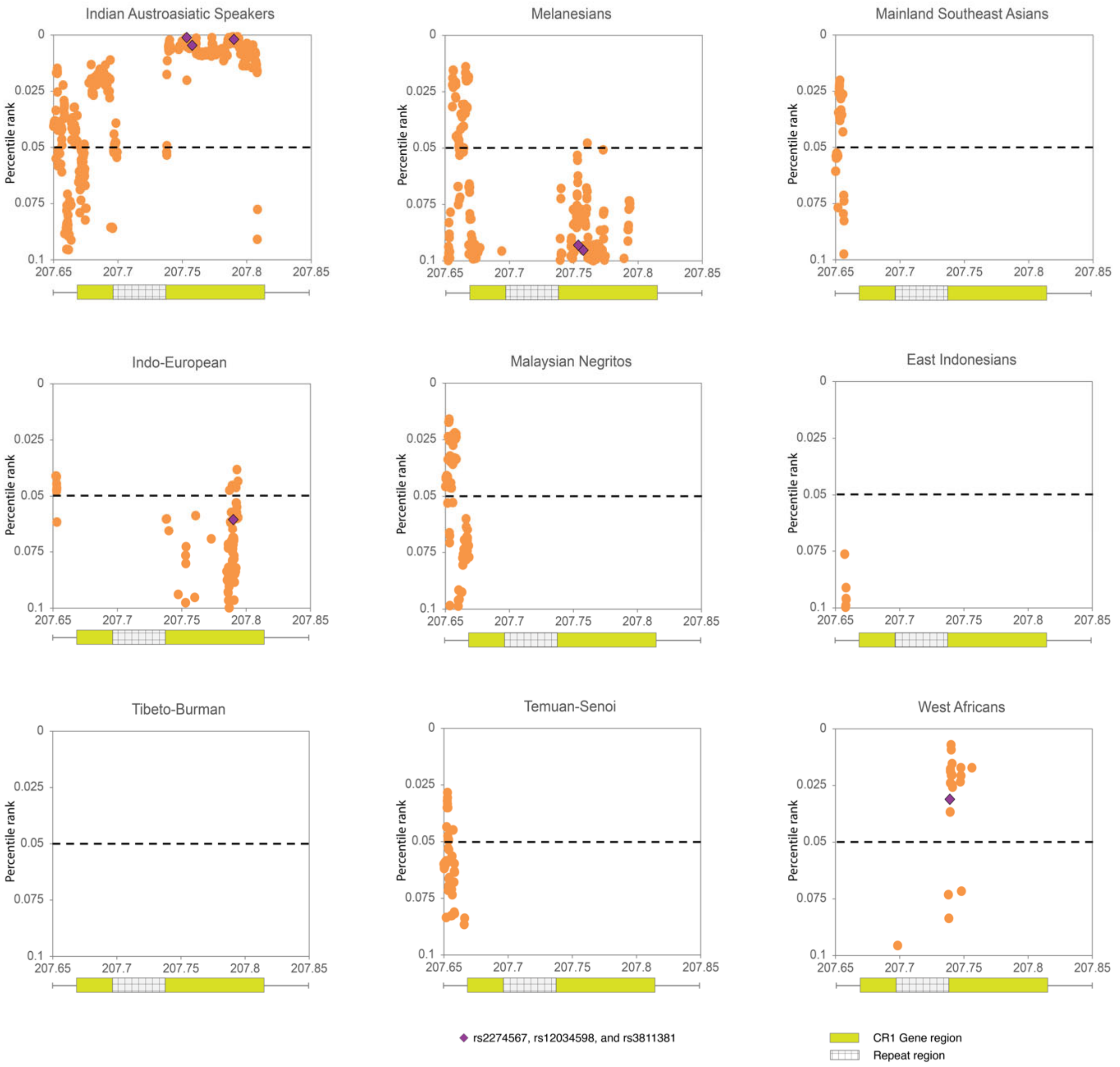

S4 Fig. Genome-wide percentile ranking of the standardised XP-EHH tests against Mongol. The XP-EHH values are plotted in the CR1 gene region including 50kb upstream and downstream for each of the 9 endemic population groups compared to the reference population of Mongols (non-endemic). Dots and triangles represent SNPs having percentile ranking values equal or lower then 0.10 (top 10%) indicated on the Y axis over the location of SNPs on chromosome 1 (X axis) in Mega bases (Mb). The green bar under the X axis represents the CR1 gene region, and the mesh area indicates repeats. The regions 50kb upstream and downstream of the CR1 gene are indicated as a line. In the DNA repeat region no SNPs called. We detected a larger number of SNPs having a percentile ranking of top 5% in the two endemic population groups (Indian Austroasiatic, Melanesians, and West Africans) in the CR1 gene region. Purple triangles indicate rs2274567, rs12034598, and rs3811381.
