# Supplementary material for: Population-specific positive selection on low CR1 expression in malaria-endemic regions
Source: PLoS One. 2023 Jan 10;18(1):e0280282. doi: 10.1371/journal.pone.0280282 (PMC9831336; doi:10.1371/journal.pone.0280282)
Supplement: S6 Fig — The branch length of endemic population group are plotted in the CR1 gene region including 50kb upstream and downstream for each of the endemic population groups versus two non‐endemic population groups, Mongols and Europeans. Dots and triangles represent SNPs having percentile ranking values equal or lower then 0.10 (top 10%) of midpoints of windows on chromosome 1 in Mega bases (Mb) on the X axis. The green bar under the X axis represents the CR1 gene region, and the mesh area indicates repeats. The regions 50kb upstream and downstream of the CR1 gene are indicated as a line. In the DNA repeat region no SNPs called. Purple triangles indicate the locations of windows containing rs2274567 exon 22, rs12034598 intron 24, and rs3811381 exon 33 SNPs. (PDF) [file pone.0280282.s006.pdf]

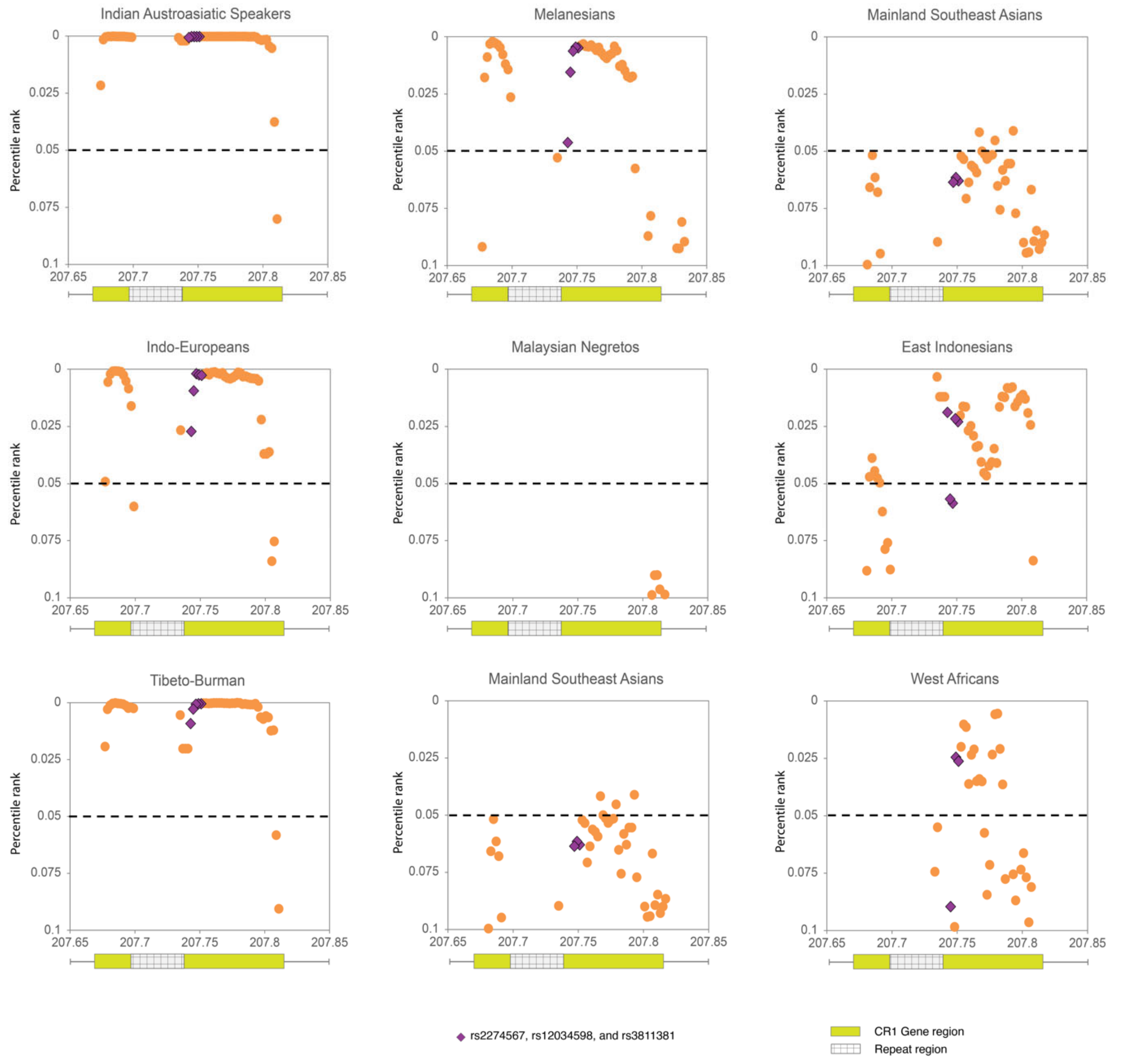

S6 Fig. Genome-wide percentile ranking of the PBS results. The branch length of endemic population group are plotted in the CR1 gene region including 50kb upstream and downstream for each of the endemic population groups versus two non-endemic population groups, Mongols and Europeans. Dots and triangles represent SNPs having percentile ranking values equal or lower then 0.10 (top 10%) of midpoints of windows on chromosome 1 in Mega bases (Mb) on the X axis. The green bar under the X axis represents the CR1 gene region, and the mesh area indicates repeats. The regions 50kb upstream and downstream of the CR1 gene are indicated as a line. In the DNA repeat region no SNPs called. Purple triangles indicate the locations of windows containing rs2274567 exon 22, rs12034598 intron 24, and rs3811381 exon 33 SNPs..
