# Supplementary material for: Population-specific positive selection on low CR1 expression in malaria-endemic regions
Source: PLoS One. 2023 Jan 10;18(1):e0280282. doi: 10.1371/journal.pone.0280282 (PMC9831336; doi:10.1371/journal.pone.0280282)
Supplement: S7 Fig — The chromosome position of the region for each tree is shown on the top of the tree. The region for the four tree encompasses from intron 27 to intron 35. The tree in panel C includes exon 33 (rs3811381). Red dots represent mutations (SNPs) assigned to a branch where the ancestral and derived allele were not flipped. The estimated coalescence time is shown on the Y axis in years. Vertical colored lines below the tree represent individuals in the five population groups. (PDF) [file pone.0280282.s007.pdf]

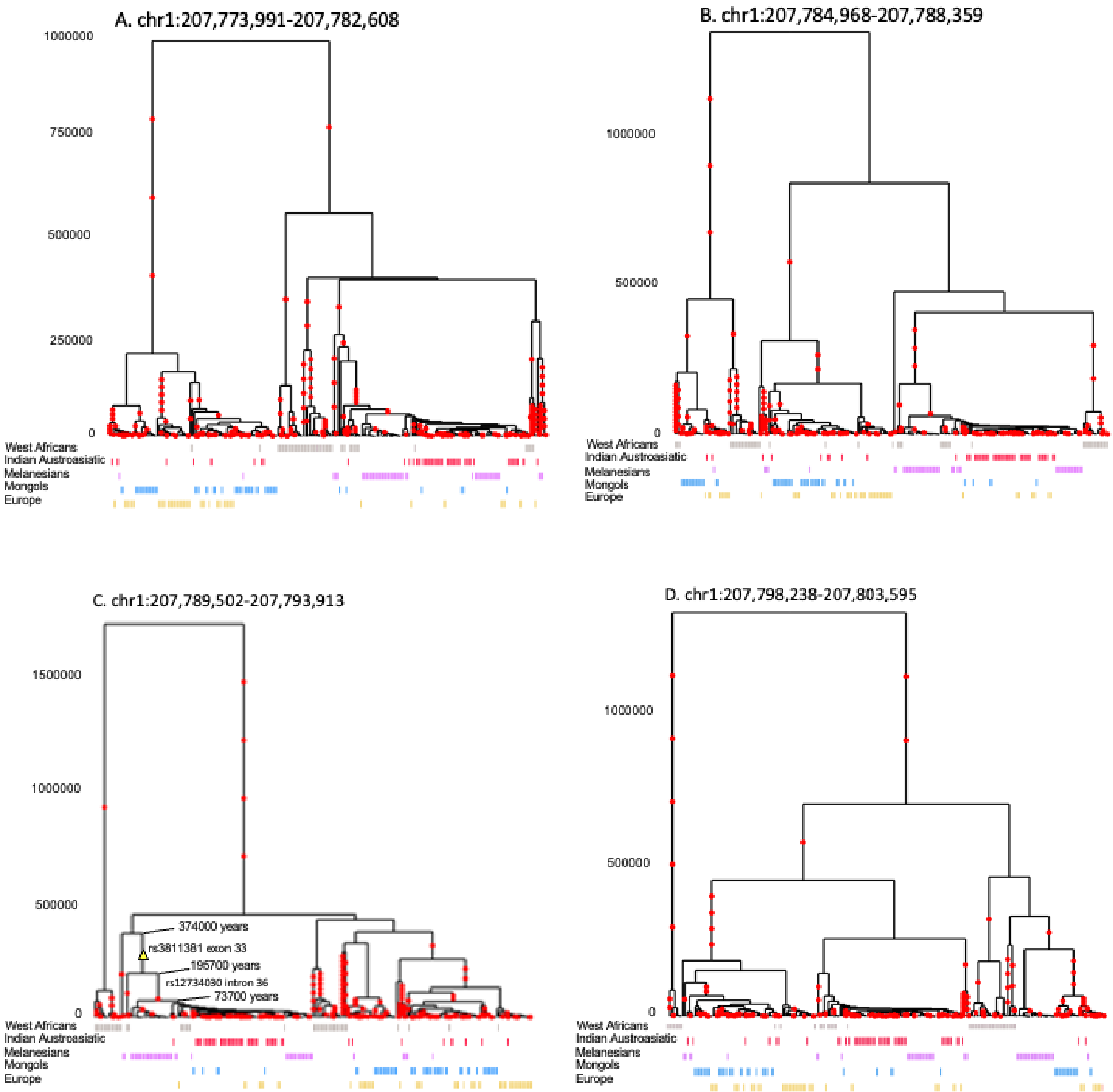

S7 Fig. Coalescence trees of the *CR1* gene region estimated by RELATE. The chromosome position of the region for each tree is shown on the top of the tree. The region for the four tree encompasses from intron 27 to intron 35. The tree in panel C includes exon 33 (rs3811381). Red dots represent mutations (SNPs) assigned to a branch where the ancestral and derived allele were not flipped. The estimated coalescence time is shown on the Y axis in years. Vertical colored lines below the tree represent individuals in the five population groups.
