# Supplementary material for: Population-specific positive selection on low CR1 expression in malaria-endemic regions
Source: PLoS One. 2023 Jan 10;18(1):e0280282. doi: 10.1371/journal.pone.0280282 (PMC9831336; doi:10.1371/journal.pone.0280282)
Supplement: S8 Fig — The estimations for the L haplogroup (A) and LS haplogroup (B) from 100 replications are plotted. The coalescence tree of Fig 3 in the main text is one of the results of this replication. Blue horizontal lines represent the time for beginning (left) and the end (right) of the branch to which the two SNPs were assigned. A red dot on the blue line indicates the middle point of the branch. The vertical black line indicates the mean of the middle points. The time was scaled by three different effective population sizes (Ne = 20000, 30000, and 40000), and a generation time of 28 years was used. (PDF) [file pone.0280282.s008.pdf]

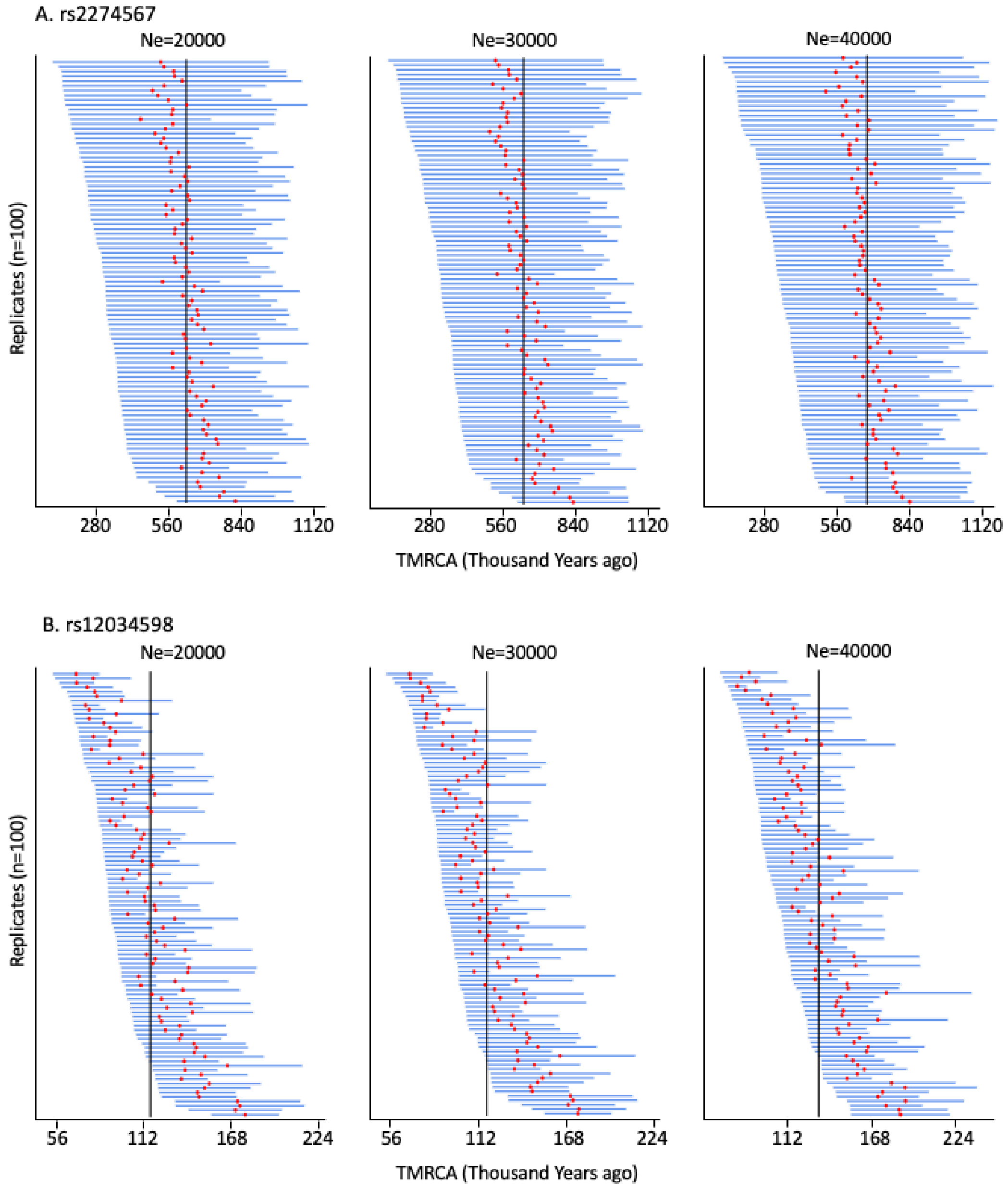

S8 Fig. Dumbbell plot of the estimation of the Time to most recent common ancestor (TMRCA). The estimations for the L haplogroup (A) and LS haplogroup (B) from 100 replications are plotted. The coalescence tree of Figure 3 in the main text is one of the results of this replication. Blue horizontal lines represent the time for beginning (left) and the end (right) of the branch to which the two SNPs were assigned. A red dot on the blue line indicates the middle point of the branch. The vertical black line indicates the mean of the middle points. The time was scaled by three different effective population sizes (Ne=20000, 30000, and 40000), and a generation time of 28 years was used.
