# Supplementary material for: Population-specific positive selection on low CR1 expression in malaria-endemic regions
Source: PLoS One. 2023 Jan 10;18(1):e0280282. doi: 10.1371/journal.pone.0280282 (PMC9831336; doi:10.1371/journal.pone.0280282)
Supplement: S9 Fig — We estimated the coalescence time for the two branches which include rs3811381 (A) and rs12734030 (B), respect, and perform the analysis 100 times. Blue horizontal lines represent the time for beginning (left) and the end (right) of each of the branches. A red dot on the blue line indicates the middle point of the branch. The vertical black line indicates the mean of the middle points. The time was scaled by three different effective population sizes (Ne = 20000, 30000, and 40000), and a generation time of 28 years was used. (PDF) [file pone.0280282.s009.pdf]

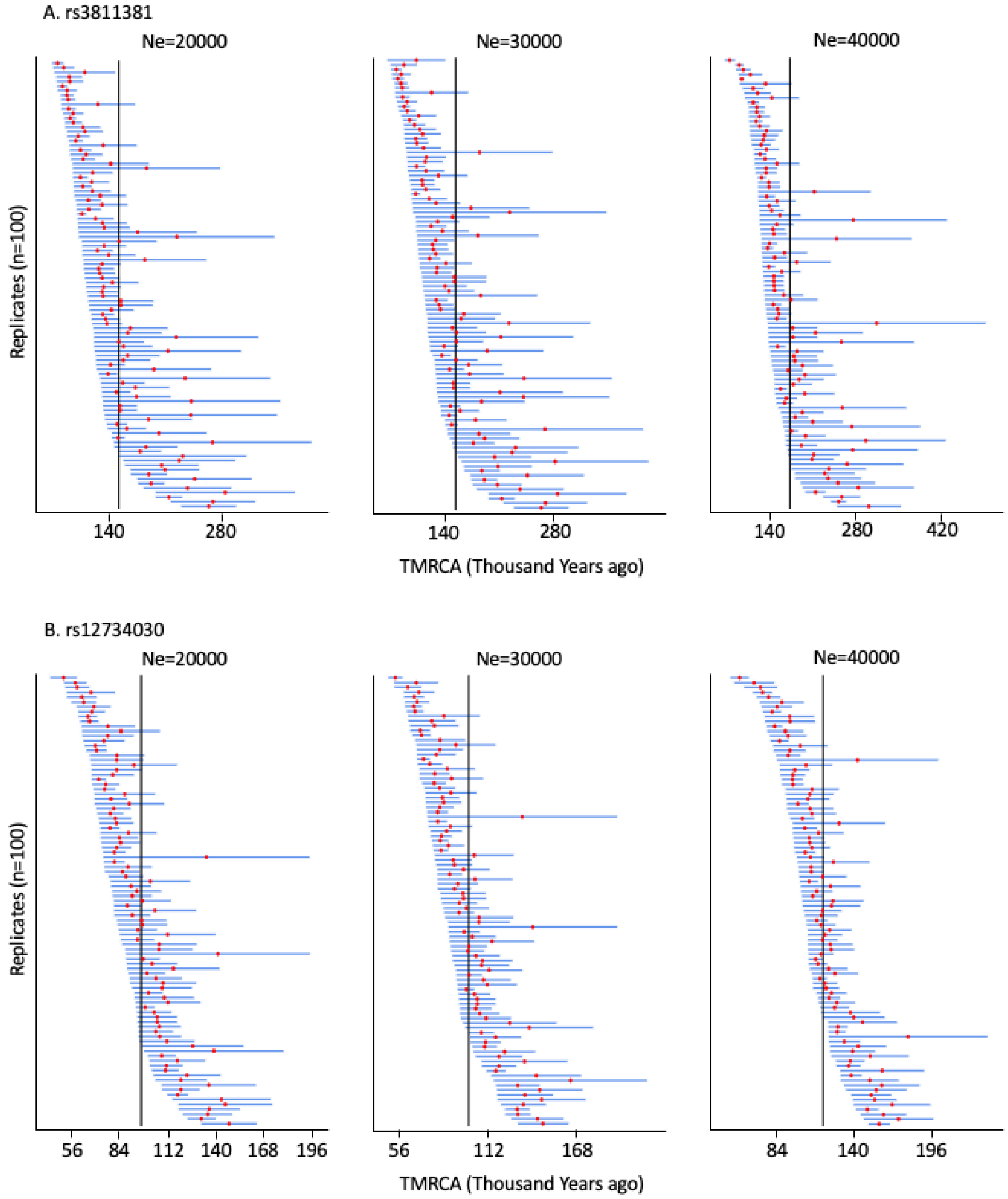

S9 Fig. Dumbbell plot of the estimation of the Time to most recent common ancestor (TMRCA) of the tree of S7C Fig. We estimated the coalescence time for the two branches which include rs3811381 (A) and rs12734030 (B), respect, and perform the analysis 100 times. Blue horizontal lines represent the time for beginning (left) and the end (right) of each of the branches. A red dot on the blue line indicates the middle point of the branch. The vertical black line indicates the mean of the middle points. The time was scaled by three different effective population sizes (*Ne* = 20000, 30000, and 40000), and a generation time of 28 years was used.
