# Supplementary material for: Population-specific positive selection on low CR1 expression in malaria-endemic regions
Source: PLoS One. 2023 Jan 10;18(1):e0280282. doi: 10.1371/journal.pone.0280282 (PMC9831336; doi:10.1371/journal.pone.0280282)
Supplement: S10 Fig — The CR1 gene expression levels in brain tissue of the six SNPs under positive selection are shown in the violin plots. Allele‐specific cis-eQTLs in human brain hippocampus tissue are retrieved from the Genotype‐Tissue Expression (GTEx Analysis Release V8 dbGaP Accession phs000424.v8.p2) database. The teal region indicates the density distribution of the samples in each genotype. The white line in the box plot (black) shows the median value of the expression of each genotype. All SNPs show significant difference (P value under the SNP rs ID) of expression level between genotypes. (PDF) [file pone.0280282.s010.pdf]

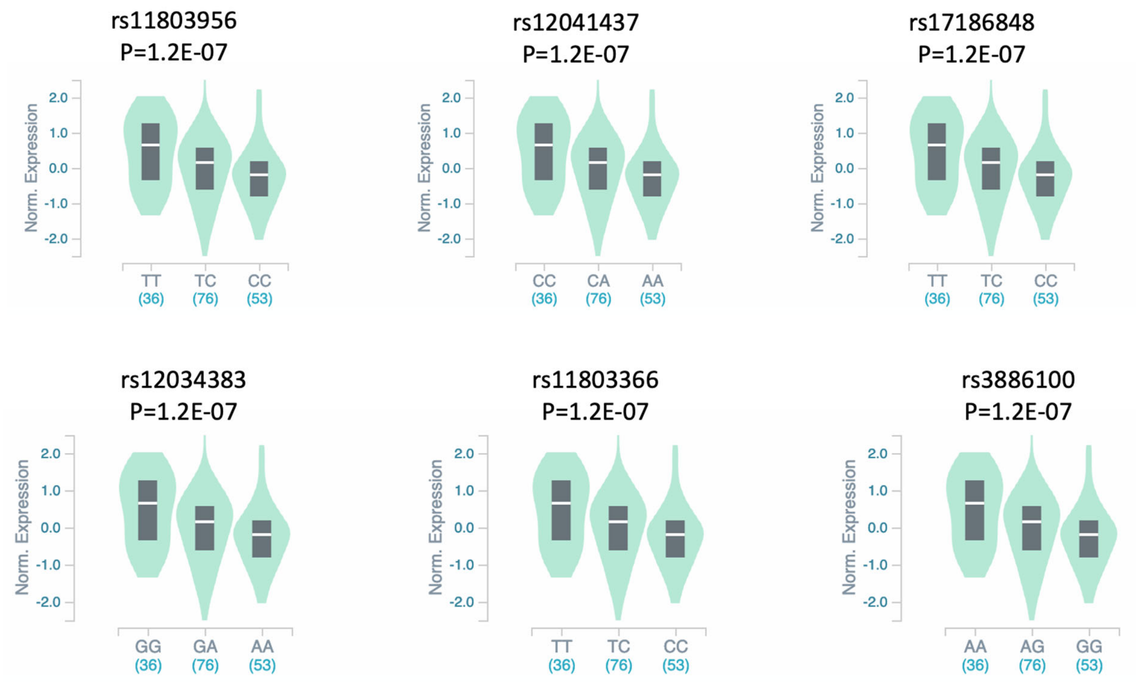

S10 Fig. CR1 expression levels in Brain tissue. The CR1 gene expression levels in brain tissue of the six SNPs under positive selection are shown in the violin plots. Allele-specific cis-eQTLs in human brain hippocampus tissue are retrieved from the Genotype-Tissue Expression (GTEx Analysis Release V8 dbGaP Accession phs000424.v8.p2) database. The teal region indicates the density distribution of the samples in each genotype. The white line in the box plot (black) shows the median value of the expression of each genotype. All SNPs show significant difference (P value under the SNP rs ID) of expression level between genotypes.
