# Supplementary material for: Population-specific positive selection on low CR1 expression in malaria-endemic regions
Source: PLoS One. 2023 Jan 10;18(1):e0280282. doi: 10.1371/journal.pone.0280282 (PMC9831336; doi:10.1371/journal.pone.0280282)
Supplement: S13 Fig — Background map indicates the malaria transmission rate in each state of the country based on the Annual Parasite Incidence (API) which denotes malaria cases per 1000 population amongst individuals of any age. API values were calculated based on the statistics of malaria cases obtained from National Vector Borne Diseases Control Programme, India. (PDF) [file pone.0280282.s013.pdf]

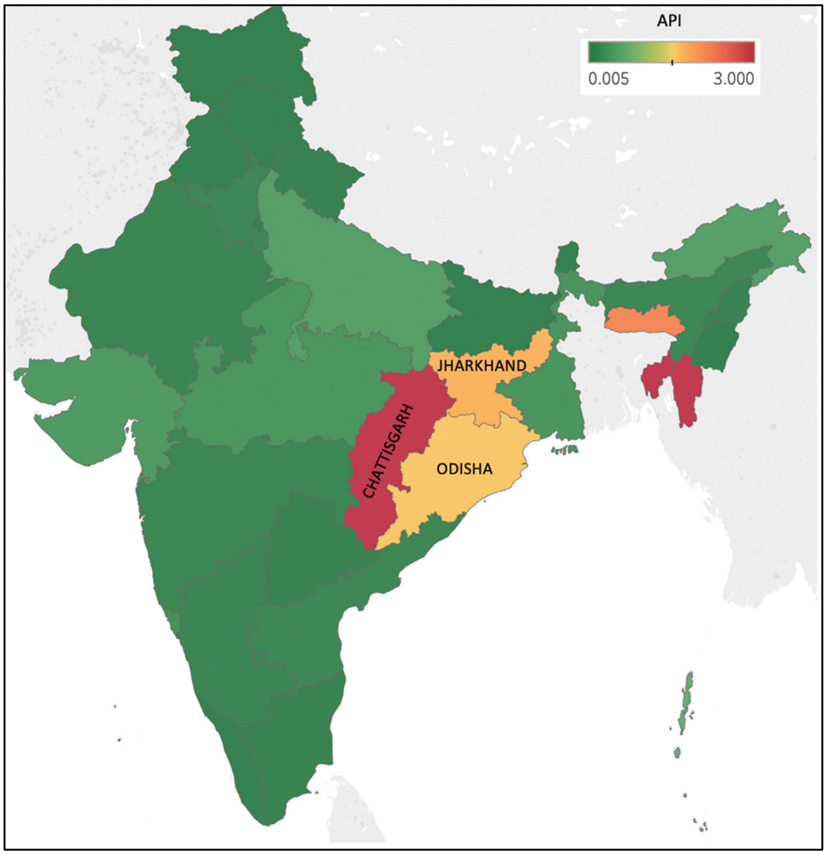


**S13 Fig. Transmission map of malaria in India.** Background map indicates the malaria transmission rate in each state of the country based on the Annual Parasite Incidence (API) which denotes malaria cases per 1000 population amongst individuals of any age. API values were calculated based on the statistics of malaria cases obtained from National Vector Borne Diseases Control Programme, India.
