# Supplementary material for: Population-specific positive selection on low CR1 expression in malaria-endemic regions
Source: PLoS One. 2023 Jan 10;18(1):e0280282. doi: 10.1371/journal.pone.0280282 (PMC9831336; doi:10.1371/journal.pone.0280282)
Supplement: Attachment — Submitted filename: CR1_RevResponses.docx [file pone.0280282.s022.docx]

Journal Requirements:

When submitting your revision, we need you to address these additional requirements.

1. Please ensure that your manuscript meets PLOS ONE's style requirements, including those for file naming. The PLOS ONE style templates can be found at

<https://journals.plos.org/plosone/s/file?id=wjVg/PLOSOne_formatting_sample_main_body.pdf> and

<https://journals.plos.org/plosone/s/file?id=ba62/PLOSOne_formatting_sample_title_authors_affiliations.pdf>

2. Thank you for stating the following financial disclosure:

“This research was supported by the Singapore Ministry of Education, Academic Research Fund Tier 1 (grant number 2017-T1-001-046). The computational work for this article was performed in part on resources of the National Supercomputing Centre, Singapore (<https://www.nscc.sg>) supported by Project 12000454.”

Please state what role the funders took in the study.  If the funders had no role, please state: "The funders had no role in study design, data collection and analysis, decision to publish, or preparation of the manuscript."

If this statement is not correct you must amend it as needed.

Please include this amended Role of Funder statement in your cover letter; we will change the online submission form on your behalf.

Response: The financial disclosure should appear as following:

“This research was supported by the Singapore Ministry of Education, Academic Research Fund Tier 1 (grant number 2017-T1-001-046). The funders had no role in study design, data collection and analysis, decision to publish, or preparation of the manuscript.”

3. Thank you for stating the following in your Competing Interests section:

“The authors declare no conflict of interest.”

Please complete your Competing Interests on the online submission form to state any Competing Interests. If you have no competing interests, please state "The authors have declared that no competing interests exist.", as detailed online in our guide for authors at <http://journals.plos.org/plosone/s/submit-now>

 This information should be included in your cover letter; we will change the online submission form on your behalf.

Response: The Competing Interests statement was removed from the manuscript and will appear in the cover letter instead as following:

Competing interests: The authors have declared that no competing interests exist.

4. In your Data Availability statement, you have not specified where the minimal data set underlying the results described in your manuscript can be found. PLOS defines a study's minimal data set as the underlying data used to reach the conclusions drawn in the manuscript and any additional data required to replicate the reported study findings in their entirety. All PLOS journals require that the minimal data set be made fully available. For more information about our data policy, please see <http://journals.plos.org/plosone/s/data-availability>.

Upon re-submitting your revised manuscript, please upload your study’s minimal underlying data set as either Supporting Information files or to a stable, public repository and include the relevant URLs, DOIs, or accession numbers within your revised cover letter. For a list of acceptable repositories, please see <http://journals.plos.org/plosone/s/data-availability#loc-recommended-repositories>. Any potentially identifying patient information must be fully anonymized.

Important: If there are ethical or legal restrictions to sharing your data publicly, please explain these restrictions in detail. Please see our guidelines for more information on what we consider unacceptable restrictions to publicly sharing data: <http://journals.plos.org/plosone/s/data-availability#loc-unacceptable-data-access-restrictions>. Note that it is not acceptable for the authors to be the sole named individuals responsible for ensuring data access.

We will update your Data Availability statement to reflect the information you provide in your cover letter.

Response: The Data Availability statement was removed from the manuscript and will appear in the cover letter instead as following:

Data Availability Statement: The datasets analyzed for this study can be found in the European Genome-phenome Archive (EGA) under accession number EGAS00001002921.

5. We note that Figures 1 and 4 in your submission contain [map/satellite] images which may be copyrighted. All PLOS content is published under the Creative Commons Attribution License (CC BY 4.0), which means that the manuscript, images, and Supporting Information files will be freely available online, and any third party is permitted to access, download, copy, distribute, and use these materials in any way, even commercially, with proper attribution. For these reasons, we cannot publish previously copyrighted maps or satellite images created using proprietary data, such as Google software (Google Maps, Street View, and Earth). For more information, see our copyright guidelines: <http://journals.plos.org/plosone/s/licenses-and-copyright>.

We require you to either (1) present written permission from the copyright holder to publish these figures specifically under the CC BY 4.0 license, or (2) remove the figures from your submission:

   a. You may seek permission from the original copyright holder of Figures 1 and 4 to publish the content specifically under the CC BY 4.0 license.

We recommend that you contact the original copyright holder with the Content Permission Form (<http://journals.plos.org/plosone/s/file?id=7c09/content-permission-form.pdf>) and the following text:

“I request permission for the open-access journal PLOS ONE to publish XXX under the Creative Commons Attribution License (CCAL) CC BY 4.0 (<http://creativecommons.org/licenses/by/4.0/>). Please be aware that this license allows unrestricted use and distribution, even commercially, by third parties. Please reply and provide explicit written permission to publish XXX under a CC BY license and complete the attached form.”

Please upload the completed Content Permission Form or other proof of granted permissions as an "Other" file with your submission.

 In the figure caption of the copyrighted figure, please include the following text: “Reprinted from [ref] under a CC BY license, with permission from [name of publisher], original copyright [original copyright year].”

   b. If you are unable to obtain permission from the original copyright holder to publish these figures under the CC BY 4.0 license or if the copyright holder’s requirements are incompatible with the CC BY 4.0 license, please either i) remove the figure or ii) supply a replacement figure that complies with the CC BY 4.0 license. Please check copyright information on all replacement figures and update the figure caption with source information. If applicable, please specify in the figure caption text when a figure is similar but not identical to the original image and is therefore for illustrative purposes only.

The following resources for replacing copyrighted map figures may be helpful:

 USGS National Map Viewer (public domain): <http://viewer.nationalmap.gov/viewer/>

The Gateway to Astronaut Photography of Earth (public domain): <http://eol.jsc.nasa.gov/sseop/clickmap/>

Maps at the CIA (public domain): <https://www.cia.gov/library/publications/the-world-factbook/index.html> and <https://www.cia.gov/library/publications/cia-maps-publications/index.html>

NASA Earth Observatory (public domain): <http://earthobservatory.nasa.gov/>

Landsat: <http://landsat.visibleearth.nasa.gov/>

USGS EROS (Earth Resources Observatory and Science (EROS) Center) (public domain): [http://eros.usgs.gov/#](http://eros.usgs.gov/)

Natural Earth (public domain): <http://www.naturalearthdata.com/>

[Note: HTML markup is below. Please do not edit.]

Response: All the satellite imagines that we have used to build the maps in Fig 1 and 4 are non-copyrighted, and created in Tableau software v. 2021.2. The original sources of the figures allow users to utilize freely the imagines under the CC BY license. We have acknowledged the original sources in the manuscript (P. 5, L. 97-98).


Reviewers' comments:

# Reviewer #1:

The novelty of this manuscript is two fold as mentioned below
One is finding of NEW SNPs related to protection of malaria and the other, more important than the first one, is finding a population-specific manner of positive selection.
Similar result for the latter case is reported by an example of LCT persistence, which shows different SNPs are target of this selection between African and European. However, such a case of population specific selection is not commonly observed.
However, in this manuscript, there are several unclear points commented as follows.
I will recommend revision of this manuscript for its publication.

Major comments:
1) This manuscript described four SNPS, rs2274567, rs3811381, rs12034598, and rs3886100. But they did not mention the latter two SNPs are novel ones to protect people from malaria, when they appeared at the first time. Later, on line 221 they mentioned “two novel variants” are rs12034598 and rs3886100. If the authors wanted to emphasize this result, description of these two SNPs would be separated the previously known ones and the authors are recommended to make a new paragraph to describe this. The current description of theses alleles may lead confusion to readers.

Response: We amended the main text in several parts in order to describe the two SNPs as novel variants associated with malaria. The Conclusions section was also amended to emphasize the two new adaptive variants, rs12034598 in Indian Austroasiatic and Melanesian populations and rs3886100 in West Africans, associated with low levels of CR1 expression (P. 16. L. 326-329).

2) TMRCA estimation (Adaptive haplotypes and their age): RELATE identified 17 haplotype blocks in the region. On line 180, “the haplotype trees show similar pattern of phylogeny, especially for the trees of the haplotype blocks containing the regions from exon2 to exon 33”. When I looked at the S7 Fig., based on the legend the trees are from intron 27 to exon 35. There is no clear correspondence of each tree with exons/introns. On line 196, “suggest LD throughout the region between the two exons” is not appropriately expressed. This should be “suggest strong LD (r2=0.89) throughout the region between the two SNPs (rs2274567 in exon 22, rs3811381 in exon 33)”.

Response: The line 180 (now P.10, L. 206-209) states that the region is from exon 22 (not exon2) to exon 33: “Across the haplotype blocks, the haplotype trees show similar patterns of phylogeny, especially for the trees of the haplotype blocks containing the regions from exon 22 to exon 33 (Fig 3 and S7 Fig).” Figure 3 includes exon 22 whereas trees in the supplementary figures include downstream regions as well.

We amended the line 196 (now P. 11, L. 234-235) as suggested.

Minor comments:
1) On page6 line 109-110: top-ranked values were selected for iHS: Significant signals for iHS appears either positive and negative. In this case the test should be both sided. If so, the top-ranked values should be 2.5% for each side. But in the present description, we can read 5% for each side because of the sentence of line 107-108. You should distinguish “one-side test” or “ both-side test”.

Response: We amended the Methods section accordingly to clarify rankings for the XP-EHH, iHS, and PBS tested performed in our study (P. 6, L. 118-120). Particularly, “For XP-EHH and PBS, we performed a “one-sided test” by taking top 5% of positive values, whereas for iHS we performed a “two-sided test” by taking top 2.5% of positive values and bottom 2.5% of negative values”.

2) On page 7 line 124: to avoid inaccurate inference. The word of “inaccurate” is vague. Please rewrite this phrase by clear expression.

Response: We amended this sentence on P. 7, L. 134-135 and provided more information.

3) On page 7 line 128-129: “for 100 different individual sets of 120 randomly sampled genomes”. This sentence may be confusing for readers. What do the authors mean “100 different individual sets”? I guess that “100 (different) sets of 120 randomly sampled genomes” is clearer.

Response: We modified the sentence on P. 7, L. 139 as suggested.

4) On page 8 line 155: “have the top 1.12% and 1.28% of the percentile rank in the whole genome”. I am not sure about “percentile rank of what” and “whose whole genome”.

Response: We amended the text on P. 9, L. 180-181 to clearly specify the percentile ranks for the two adaptive variants, rs2274567 and rs3811381, in the iHS tests on the Indian Austroasiatic of which method and in which populations.

5) On page 9 line 182-183: “defined by six SNPs that include rs2274567. Thus, we designated the two haplogroups as low(L) and high(H) expression level haplogroups”. If the authors use “Thus”, the authors should mention that rs2274567 is involved in the expression level of CR1.

Response: We amended the text as suggested (P. 10, L. 209).

6) On page 10 line 186: "the most frequent subnode". For me this expression reads somewhat strange. This phrase may be “the most frequent subclade”.

Response: We amended the text as suggested (P. 10, L. 213).

7) On page 10 line 192-193: "based on 100 repeated estimates of random sampling". First, Di this mean that this estimation based on 100 bootstrap resampling? Second, resampling is from the total samples or from only L or LS haplogroups.

Response: We meant 100 bootstrap resampling of the total samples. We amended the text to make it clear (P. 11, L. 230-231).

8) On page 10 line 204: Do the authors mean “minimum selection constraints” as “relaxation of functional constraints” or “minimum selection coefficients”?

Response: We mean no significant evidence of selection. We modified main text to make it clearer (P. 12, L. 242).

9) On page 12 line 238,239,241: The frequency of LS 75% to 100% does not seem to agree to 9.6 per 100,000 people (0.0096%). What is the base of this LS frequency? The same argument is for line 239 and 241.

Response: As first value inside the brackets, we meant the LS frequency, whereas as a second value, we indicated malaria mortality rate. We amended the main text accordingly to distinguish the two types of information (P. 13, L. 276-279).

# Reviewer #2:

Despite CR1 gene has been extensively studied to be in association with rosseting of red blood cells that subsequently cause microvascular obstruction and eventual severe/cerebral malaria, the complete spectrum of variations across the whole gene and its flanking regulatory regions are remained unknown. This work harnesses the power of next generation sequencing and has sequenced the complete CR1 gene and identify all variations from individuals residing in malaria-endemic and non-endemic regions. Further analyses of positive selection identified SNPs that could act as protective biomarkers against malaria infection / development toward severe/cerebral malaria.

A number of comments on this manuscript are as follow:

1. Previous studies always select a few SNPs of CR1 gene for testing its association to malaria infection. You pointed out that the association could be contrasting. Thus, this should be overcome. In addition, as a gene could have a few hundred variations across human populations, the power of sequencing and subsequent analyses should be harnessed to select candidate SNPs with strong protective effects. Please have an in-depth literature review over this and articulate the specific objective you wish to work out.

Response: As written in the introduction (P. 3-4, L. 48-64), we made a very in-depth literature research which describes in detail the associations of the L and H alleles of the *CR1* gene with the protection or susceptibility against malaria in several human populations. This literature research includes 15 citations (refs. 13-28). From this research we have observed that sometime the high expression of the *CR1* gene (H allele) was reported to be associated with malaria protection, despite the low expression (L allele) should be the one conferring protection. Our objective was to clarify this discrepancy and report the associations in the same ethnic groups by utilizing whole genome sequencing data and methods of population genetics which should provide stronger evidence of whether the low expression or the high expression is the one that is conferring protection. We have described in detail results of this objective in our results section.

2. Do you specify that the force of selection is the lethal cerebral malaria or severe types of malaria (in exception to cerebral one). Some species might be more pathogenic and lethal e.g. P. falciparum infection commonly lead to cerebral malaria, attributed to different pathogenesis pathways. And the distribution of the different species could be different, e.g. P. knowlesi in ISEA. In addition, using malaria case numbers as a measure of endemicity and force of selection is an appropriate proxy, but unfortunately these case numbers do not specify which species of Plasmodium that caused which severe types of malaria. Judging on that, these could serve to drive the selection force differently. Looking at the high number of cases in the current days, I wonder has this force of selection yet been fixed, and thus the method of analysis should be revised? Please clarify these inter-related problems in detail.

Response: We thank you the reviewer to highlight this very important point on the different pathogenesis and prevalence of the Plasmodium species. In our Fig 4, we have reported epidemiology of malaria mortality caused by P.falciparum species which as mention is the main cause of cerebral malaria. As such, the fact that we see high frequencies of the *CR1* LS haplogroup in the same regional areas where the *P. falciparum* is linked to high mortality, and it is highly widespread is in agreement with the protective role of the LS against that species. Indeed, cerebral malaria can be caused as well by other species such as *P. vivax,* but very rarely as compared to *falciparum*. *P. vivax* is more widespread in the west areas where we did not detect a high frequency of the *CR1* LS haplogroup (Fig 4). On contrary, *P. falciparum* is highly widespread in the east where we detected high frequencies of the LS haplogroup (Fig 4). As such, we concluded that the main driving force of selection is coming from the *P. falciparum* species, which is linked to cerebral malaria pathogenesis. For all these reasons, we believe that the methods used in this research is sound and appropriate to identify adaptive variants against of malaria. We mention the cause of cerebral malaria is P. falciparum in discussion (P. 13, L. 275 and P.14, L. 302).

3. Since you inferred that the positive selection already occurred before human migrating out of Africa, I suppose that majority of the extant human racial groups should carry the same polymorphic SNPs which are almost fix, in both endemic and non-endemic extant populations. But your data did not find so. What could be the reason?

Response: We did not infer that positive selection occurred before the out-of-Africa migration, but we stated that the L allele were present before the migration and both L and H alleles have maintained in human populations because there is no selection pressure in either of the alleles. After the out-of-Africa migration and settlement of populations in malaria endemic region, the L allele has become adaptive. Thus, the selection occurred in population-specific manner. To clarify this point, the main text was amended (P. 13, L. 266).

4. Some samples are carrying more variations in the gene. How do you test and remove the mentioned ‘less admixed’ individuals? How do you affirm that the positively selected variants are not due to ancestry / anthropology, instead of natural selection?

Response:

1) We would like to clarify that we did not remove less admixed populations/individuals, but we removed the highly admixed ones. For example, initially, to select the endemic and non-endemic populations included in this study, we removed highly admixed populations from the GA100K dataset, based on genetic ancestry analysis such as admixture analysis. In addition, for the TMRCA analysis as stated on P. 7, L. 133-135, to build the trees we selected less admixed population groups to have accurate assessment.
2) We believe that the SNPs detected under selection in our study are not due to differences in ancestries since we performed a genome-wide ranking analysis in each population group of similar genetic ancestry. And we compared only within the population groups across whole genome.

5. Based on the GTEX paper published in 2020, a great majority of 85% of the dataset are of European American. Since European countries are not in the Malaria endemic region, and you also found that there is no signal of selection on CR1 gene among the European samples, you thereby used this public gene expression data (low CR1 expression) as your strong support that infers that this gene is also expressed low among the other multi-racial / ancestries individuals in Asia & Africa. Unless it is tested empirically by any measure of gene expression using RNA from your studies samples, this inference is invalid.

Response: Thanks for indicating the point that the expression data measured by GTEX is based on European populations. We mentioned that the expression data we are referring to was mainly reported for Europeans (P.12, L. 249).

6. The outcome of this work is derived from bioinformatic analyses of individuals with unknown history of malaria infection, and thus the obtained genotypic and allelic frequencies should represent the general polymorphisms in each regional population. However, the conclusion can only be made after a well-designed case-control association test is conducted. As such, your previous work (Gusareva et al., 2021) should have already identified similar results as this current manuscript. I wonder why CR1 analysis was split out from the previous work?

Response: Our previous study indeed mentioned the selection pressure on the *CR1* gene. In the present research, we have shown the fine-resolution history of positive selection in the *CR1* and identified novel protective variants against malaria in Asians as well as in West Africans that is reported for the first time. Our analysis of the haplogroups (Fig 3 and 4) indicate the age of the protective haplotype and its association with not only the low CR1 expression level but also slow rate of erythrocyte sedimentation. This fine-resolution analysis with genome sequencing dataset can show the population-specific manner of positive selection and convergent evolution especially for understudied Asian populations. This extensive analysis was conducted in follow-up the initial pilot screening reported by Gusareva et al., 2021.

7. The conclusion states that the current findings could be helpful in precision medicine of malaria medical management. Unfortunately, this bioinformatic findings have yet to be extensively tested but the authors already gave strong conclusive statements. I find this misleading and should be removed.

Response: We tone down our conclusions regarding the medical applications of our findings and amended the Conclusions section accordingly (P. 16).
